# Antibodies Responses to SARS-CoV-2 in a Large Cohort of Vaccinated Subjects and Seropositive Patients

**DOI:** 10.3390/vaccines9070714

**Published:** 2021-07-01

**Authors:** Emanuele Amodio, Giuseppina Capra, Alessandra Casuccio, Simona De Grazia, Dario Genovese, Stefano Pizzo, Giuseppe Calamusa, Donatella Ferraro, Giovanni Maurizio Giammanco, Francesco Vitale, Floriana Bonura

**Affiliations:** Department of Health, Promotion, Mother and Child Care, Internal Medicine and Medical Specialties, University of Palermo, Piazza delle Cliniche 2, 90127 Palermo, Italy; giuseppina.capra@unipa.it (G.C.); alessandra.casuccio@unipa.it (A.C.); simona.degrazia@unipa.it (S.D.G.); dario.genovese@unipa.it (D.G.); stefano.pizzo@unipa.it (S.P.); giuseppe.calamusa@unipa.it (G.C.); donatella.ferraro@unipa.it (D.F.); giovanni.giammanco@unipa.it (G.M.G.); francesco.vitale@unipa.it (F.V.); floriana.bonura82@gmail.com (F.B.)

**Keywords:** COVID-19 vaccine, SARS-CoV-2 infection, antibody concentrations

## Abstract

COVID-19 is a current global threat, and the characterization of antibody response is vitally important to update vaccine development and strategies. In this study we assessed SARS-CoV-2 antibody concentrations in SARS-CoV-2 positive patients (N = 272) and subjects vaccinated with the BNT162b2 m-RNA COVID-19 vaccine (N = 1256). For each participant, socio-demographic data, COVID-19 vaccination records, serological analyses, and SARS-CoV-2 infection status were collected. IgG antibodies against S1/S2 antigens of SARS-CoV-2 were detected. Almost all vaccinated subjects (99.8%) showed a seropositivity to anti-SARS-COV-2 IgG and more than 80% of vaccinated subjects had IgG concentrations > 200 AU/mL. In a Tobit multivariable regression analysis, SARS-CoV-2 vaccination was statistically significantly associated with increased IgG concentrations (β coef = 266.4; *p* < 0.001). A statistically significant reduction in SARS-CoV-2 IgG concentrations was found with older age (β coef = −1.96 per year increase; *p* < 0.001), male sex (β coef = −22.3; *p* < 0.001), and days after immunization (β coef = −1.67 per day increase; *p* < 0.001). Our findings could support the vaccination campaigns confirming the high immunogenicity of the SARS-CoV-2 vaccine under investigation with respect to the natural infection. Further studies will be required for evaluating the role of age and days after immunization in the persistence of vaccine antibodies and protection from the disease.

## 1. Introduction

Coronavirus Disease 2019 (COVID-19) is an infectious disease caused by Severe Acute Respiratory Syndrome Coronavirus 2 (SARS-CoV-2) [1]. It was reported for the first time in Wuhan (China) in December 2019 [2,3,4] and, from that time, it spread globally, resulting in a pandemic.

As of 25 March 2021, COVID-19 had affected more than 125 million patients worldwide [5]. As of the same date, in Italy, about 3,500,000 people had been infected with SARS-CoV-2 and more than 107,000 deaths were reported [5].

Clinically, SARS-CoV-2 infection might pass asymptomatically or there might be symptoms that evolve into a mild, moderate, severe, or critical disease [6]. Since the COVID-19 outbreak began, the scientific community has put a lot of effort into containing this emerging pandemic. A range of clinical and policy interventions have been implemented in order to mitigate SARS-CoV-2 spread; a better understanding of the dynamics and determinants of humoral immunity to this virus and to the new available vaccines could represent a major piece in the puzzle in the fight against the virus.

Rapid spread of SARS-CoV-2 in the population is facilitated by direct transmission through droplets and the abundancy of Angiotensin-converting enzyme 2 (ACE 2) positive cells in the nasal and pharyngeal mucosa. The spike–ACE interaction between the viral spike (S) protein and ACE2 is responsible for the initiation of the infectious process. The structural spike protein exists in the virus envelope as a homotrimer consisting of S1 and S2 subunits. The S1 subunit contains the receptor binding domain (RBD) while the S2 subunit includes the fusion peptide (FP). The RDB is the major target for neutralizing antibodies (Nt); however, the S2 subunit is also a potential target of Nt. The spike protein is also a target for the T cell response [7].

Characterization of the human antibody response to SARS-CoV-2 infection and vaccination can be vitally important for updating vaccine development and strategies generally and with the emergence of new virus variants.

In this sense, several studies have investigated the dynamics of the SARS-CoV-2 infection’s humoral response, focusing on dynamic changes in serum IgM and IgG [8,9] or immunological memory for fixed time ranges [10,11]. The main object of these studies has been the immune defense against the spike (S) protein [11,12,13,14].

Several studies have evaluated the humoral response to anti-SARS-CoV-2 vaccines, although usually these data referred to samples with a small size and investigated relatively short time frames after second dose vaccination [15].

Despite the efforts made by the international scientific community, to date there is a paucity of studies that compare the humoral responses consequent to natural and artificial immunity. According to the previous considerations, in the current study we aimed to assess SARS-CoV-2 antibody concentrations in a large cohort of subjects including both SARS-CoV-2 positive patients and vaccinated people. Moreover, we evaluated the serological response with respect to some potential confounding factors such as age, sex, and time since immunization occurred.

## 2. Materials and Methods

This was an observational cross-sectional study that involved two different cohorts of individuals who consecutively underwent a serological analysis at the A.O.U.P. “P. Giaccone” Hospital of Palermo (Italy). A first cohort (Vaccination Cohort) has included individuals who received the first dose of the BNT162b2 m-RNA COVID-19 vaccine between 28 December 2020 and 7 February 2021 and the second dose of the same vaccine between 18 January 2021 and 1 March 2021. The second cohort (SARS-CoV-2 Cohort) included patients with a previous confirmed diagnosis of SARS-CoV-2 infection. Patients had decided independently to be tested for anti-SARS-CoV-2 in order to check seroconversion after vaccination or natural infections. At the time of blood withdraw they were asked for consent to participate in the study and were enrolled.

To meet the aims of the study, patients who met the following criteria were considered in the statistical analyses:

- Aged between 18 and 65 years;
- Underwent a serological analysis between 10 and 60 days after SARS-CoV-2 positivity (for the SARS-CoV-2 Cohort) and had not been vaccinated against SARS-CoV-2;
- Underwent a serological analysis between 10 and 60 days after the first dose vaccination (for the Vaccination Cohort) and did not have a positive anamnesis for SARS-CoV-2.

For each patient, the following information was collected:

- for the Vaccination Cohort: age, sex, date at first dose vaccination, date at second dose vaccination, date at serological analyses;
- for the SARS-CoV-2 Cohort: age, sex, date at serological analyses, date at first positive SARS-CoV-2 molecular test, worst clinical outcome (codified as asymptomatic, mild, moderate, severe, critical, deceased). SARS-CoV-2 positivity was confirmed by searching for the notification status in the national database, updated daily, by the local health authorities and provided by the Istituto Superiore di Sanità (ISS).

All vaccinated subjects had vaccinated with the BNT162b2 m-RNA COVID-19 vaccine and vaccine administration was performed according to the guidelines provided by the Italian Medicines Agency—AIFA [16].

The study was approved by the Ethical Committee of the A.O.U.P. “P. Giaccone” on June 24th, 2020, protocol number 0006. All patients expressed a formal consent to the blood withdrawal in accordance with the national law.

### 2.1. Laboratory Analyses

Serum samples were analyzed by chemiluminescent immunoassay (CLIA) technology, (LIAISON^®^ SARS-CoV-2, Diasorin, Saluggia (VC)—Italy) according to the manufacturer’s instructions on the LIAISON^®^ XL Analyzer. IgG antibodies against S1/S2 antigens of SARS-CoV-2 were detected in a semi-quantitative assay with a lower limit of detection (LoD) of 0.3 AU/mL (arbitrary units/mL) and an upper limit for quantitative evaluation at 400 AU/mL. As suggested by the manufacturer, samples were considered positive when AU/mL (arbitrary unit/mL) was ≥15, and negative when AU/mL was ≤12 AU/mL, while with results between 12 and 15 AU/mL samples were considered borderline [17].

### 2.2. Statistical Analyses

Normality distribution of quantitative variables was assessed by the Shapiro–Wilk test and, accordingly, all variables that were normally distributed have been summarized as mean (SD) whereas non-normally distributed variables have been presented as median and interquartile range (IQR).

Categorical variables have been summarized as absolute number (percentage). The chi-square test was used to assess the differences in the distribution of proportions by group and the chi-square for trend was used for evaluation trends in frequencies. The Mann–Whitney rank sum test was used to compare non-parametric continuous variables including SARS-CoV-2 IgG (AU/mL) concentrations.

Since anti-SARS-CoV-2 IgGs were right censored (at levels of >400 AU/mL), for the multivariable analysis we used a Tobit linear regression (package AER available for R software). The multivariable Tobit model was built to determine the association between independent variables as age, sex, vaccination, and days after vaccination or SARS-CoV-2 positivity and SARS-CoV-2 concentrations. Results were reported as β coefficients with standard error and *p* values.

All statistical tests were two-tailed, and statistical significance was defined as *p*  ≤  0.05. Analyses were performed using R Software analysis 3.6.1. [18].

## 3. Results

The general characteristics of the two study cohorts are reported in Table 1. Overall, 1256 subjects were included in the Vaccination Cohort and 272 in the SARS-CoV-2 Cohort. The two cohorts were statistically significantly different for gender distribution (54.8% females in the Vaccination Cohort vs. 42.3% females in the SARS-CoV-2 Cohort; *p* < 0.001).

A statistically significantly difference was found among the distribution of IgG concentrations between the two cohorts with more than 80% of vaccinated subjects having concentrations above 200 AU/mL, whereas more than 80% of SARS-CoV-2 subjects had IgG concentrations below 100 AU/mL. According to the worst clinical presentation, a large majority of subjects included in the SARS-CoV-2 Cohort were asymptomatic to mild (243; 89.3%), whereas 25 (9.2%) were moderate and 4 (1.5%) were severe/critical (data not shown in the table).

**Figure 1:**
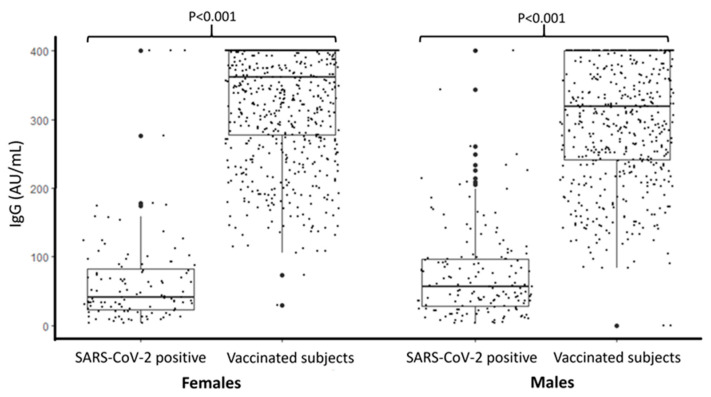
IgG concentration (AU/mL) in subjects positive for SARS-CoV-2 and in vaccinated subjects stratified by sex (LOD < 3.8 and >400 AU/mL).

**Figure 2:**
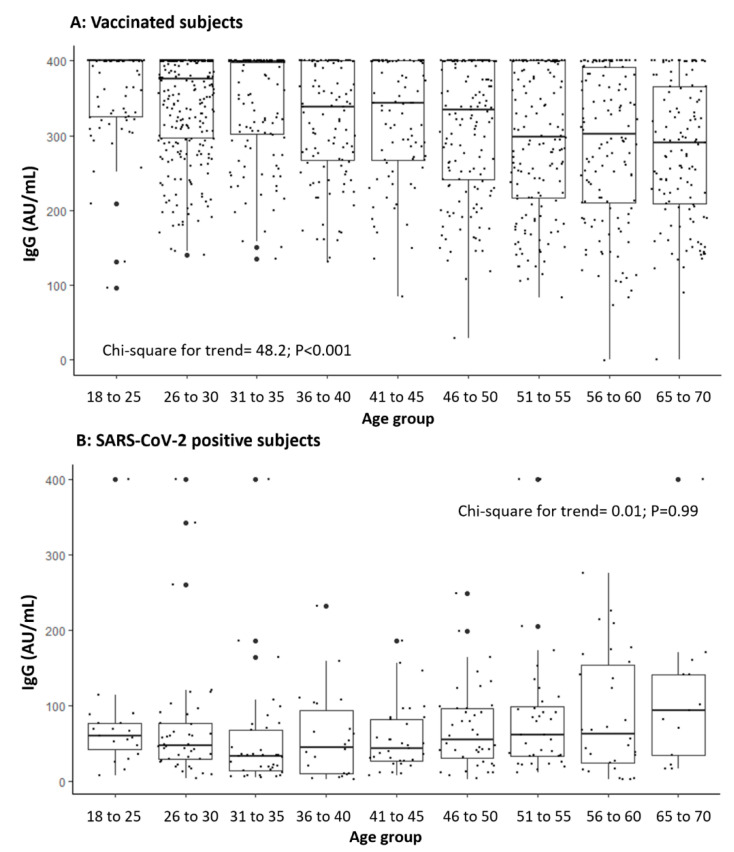
IgG concentration (AU/mL) in vaccinated subjects (**A**) and in those positive for SARS-CoV-2 (**B**) according to the age group (LOD <3.8 and >400 AU/mL).

Figure 1 shows that the IgG concentrations were statistically significantly higher among vaccinated subjects, and this difference was maintained in both sexes. In Figure 2, IgG concentrations are compared according to the different age groups of the two cohorts. A statistically significant decreasing trend was found between frequency of IgG concentrations > 400 AU/mL among vaccinated subjects and an increase of age (chi-square = 48.2; *p* < 0.001).

Table 2 summarizes the results of a Tobit regression analysis evaluating factors involved in determining the levels ofanti-SARS-CoV-2 IgGs concentrations. Overall, SARS-CoV-2 vaccination was statistically significantly associated with increased IgG concentrations (β coef = 266.4; *p* < 0.001), whereas a statistically significant reduction in anti-SARS-CoV-2 IgGs concentrations was found with older age (β coef = −1.96 per year increase; *p* < 0.001), male sex (β coef = -22.3; *p* < 0.001), and days after immunization (β coef = −1.67 per day increase; *p* < 0.001).

## 4. Discussion

Humoral response against SARS-CoV-2 is one of the key aspects for understanding both the viral clearance and vaccination effectiveness. In this study we analyzed two different cohorts of patients in order to understand the difference, if present, of antibody response due to natural or artificial immunity.

The results of the study must be carefully discussed. The first important finding is that almost all subjects vaccinated with the BNT162b2 m-RNA COVID-19 vaccine obtained an immunological response with IgG concentrations higher than those observed in patients who had natural infection, according to Phase 1/2 trial studies on immunogenicity [19]. This consideration seems to suggest that a significant difference can be found between immunological answers according to the two different exposure pathways. As reported in a recently published review, the majority of subjects who experience symptomatic SARS-CoV-2 infection develop a detectable specific antibody response in the acute phase [20]. This immunity response may be of lower magnitude in milder cases, and this should be considered when reviewing our results since in our study, a large number of patients who had SARS-CoV-2 infection were asymptomatic or pauci-symptomatic. However, it should also be stressed that they are representative of the general population aged 18 to 65 years in which mild infections represent the most common presentation of the viral infection [21]. The antibody response has also been associated with age and sex [22,23,24], and this was the main reason why we restricted inclusion criteria for the study and performed a multivariable analysis in order to check for these possible confounding factors. In addition, in the multivariable Tobit regression analysis, vaccination was found to be strongly associated with a humoral response clearly higher than that in subjects who naturally acquired the SARS-CoV-2 infection. Considering that about 80% of these latter patients had IgG concentrations below 100 AU/mL, vaccination increases significantly the probability of having higher antibody concentrations with respect to those from natural infection. These findings deserve further considerations in relationship to the possibility of administrating a single vaccine dose to seropositive subjects [11], considering this dose as a boosting of the anti-SARS-CoV-2 immune response. A similar approach has been proposed by several authors who have observed that in seropositive participants, a single dose of mRNA vaccine elicited postvaccination antibody concentrations that were similar to or exceeded concentrations found in seronegative participants who received two vaccine doses [25].

As other factors involved in the humoral response, we observed that older age, male sex, and days after immunization (natural or artificial) seem to be associated with a decrease in the probability of having higher antibody titers. Some authors have observed that patients infected with SARS-CoV-2 tend to have higher concentrations with age [26,27,28], although this evidence cannot be considered conclusive [20] since this finding could be due to the higher chance of having a severe disease in older subjects. Considering the role of the timing, it has been well documented that the kinetics of the antibody response to SARS-CoV-2 follow typical immunological paradigms with a peak around two to five weeks following disease onset and a succeeding decline [20].

All the previous considerations should be evaluated in the light of some possible limitations of the study.

First of all, humoral immune responses should be considered as correlates of protection against COVID-19 [11] and, thus, antibody levels may not be sufficient to accurately predict the infection risk. A protection due to antibody presence seems to be confirmed by experimental studies carried out on non-human primates showing protection from reinfection [29,30]. Moreover, a study conducted in the UK demonstrated that being seropositive to SARS-CoV-2 through natural infection provides robust protection from asymptomatic and symptomatic reinfection [31]. Thus, infection and the development of an antibody response could provide similar or even better protection than do the currently used SARS-CoV-2 vaccines, although antibody responses induced by SARS-CoV-2 infection are often lower in titers than those induced by vaccination. In this sense, several other studies demonstrated that infection does protect against reinfection, probably in an antibody-dependent manner [32,33,34,35].

Although it is generally accepted that high-concentration antibodies are optimal for protective immunity after SARS-CoV-2 exposure [36], it cannot be excluded that a protection can be acquired in their absence or that infection can occur when high levels are present. In this sense, establishing antibody titers as a correlate of protection and defining a protective titer should be the priorities for future studies that investigate protection provided by natural infection or vaccination.

Moreover, it should be noted that the SARS-CoV-2 S1/S2 IgG assay used in this study to detect neutralizing IgG antibodies has shown a positive agreement with the plaque reduction neutralization tests (PRNT) titers and with SARS-CoV-2 spike-binding neutralizing antibody titers [17,37], although some other authors suggest a poor direct correlation between antibody titers and neutralizing activity levels in naturally infected subjects [38].

However, although some studies observed that spike IgG concentrations are durable [11], with modest declines after 6–8 months post-symptoms onset (PSO) [10], the kinetics of humoral response over a longer time period should be evaluated in order to obtain information on the long-term immunity in both vaccinated and SARS-CoV-2 infected subjects and ascertain possible correlations between neutralizing antibodies concentrations defined by PRNT and the presence of clinical signs of illness. This could be of interest in relation to the increased circulation of SARS-CoV-2 variants.

Finally, days after COVID-19 positivity should be interpreted with caution since, in SARS-CoV-2 patients, they could underestimate the real time from the first contact with the virus.

## 5. Conclusions

Even though the study’s limitations cannot be excluded, to the best of our knowledge, this is the first study that has investigated the humoral response in a large cohort of vaccinated subjects and seropositive patients. We are confident that these findings support the vaccination campaigns, confirming the high immunogenicity of the SARS-CoV-2 vaccine under investigation with respect to that of natural infection.

## Figures and Tables

**Table 1:** Characteristics of the two cohorts of patients included in the study.

Investigated Variables	Categories	Vaccination Cohort (SARS-CoV-2 Negative)	SARS-CoV-2 Positive Cohort (Not Vaccinated)	*p*-Value
				
Total, N (%)		1256 (82.2)	272 (17.8)	
				
Age, median (IQR)		43 (30–54)	43 (31–51)	0.62
				
Gender, N (by column %)				
	- F	688 (54.8)	115 (42.3)	<0.001 *
	- M	568 (45.2)	157 (57.7)	
				
Days after first dose of vaccination or COVID positivity, median (IQR)		37 (34–41)	40 (32–49)	0.13
				
IgG (AU/mL) median (IQR)		342 (259–400)	48.9 (25.6–92.1)	<0.001
Worst clinical presentation, IgG median (IQR)				
	- Asymptomatic, Mild	-	46.4 (24–88.3)	
	- Moderate	-	90.5 (53.7–92.7)	
	- Severe, Critical	-	101.2 (22.3–191.7)	
				
IgG (AU/mL), N (by column %)				
	-Seronegative	2 (0.2)	28 (10.3)	<0.001 *
	- 12 to 15	0 (0)	10 (3.7)	
	- >15 to 50	1 (0.1)	101 (37.1)	
	- 51 to 100	7 (0.6)	77 (28.7)	
	- 101 to 150	53 (4.2)	25 (9.2)	
	- 151 to 200	90 (7.2)	15 (5.5)	
	- 201 to 250	130 (10.3)	6 (2.2)	
	- 251 to 300	184 (14.6)	2 (0.7)	
	- 301 to 350	193 (15.4)	1 (0.4)	
	- 351 to inf	596 (47.4)	6 (2.2)	

* The *p*-value refers to a statical difference within the entire group.

**Table 2:** Multivariable Tobit regression analysis of factors involved in determining SARS-CoV-2 IgG concentration.

Investigated Variables	Coefficient	Standard Error	*p*-Value
**- Vaccination (ref. COVID-19 positivity)**	266.4	7.18	<0.001
**- Female sex (ref. male)**	−22.3	5.7	<0.001
**- Age in years (per unit increase)**	−1.96	0.22	<0.001
**- Days after vaccination or positivity to COVID-19 (per unit increase)**	−1.67	0.37	<0.001

## Data Availability

The data are available under reasonable request to the corresponding authors.
